# Effect of Photodynamic Therapy with Chlorin e6 on Canine Tumors

**DOI:** 10.3390/life12122102

**Published:** 2022-12-14

**Authors:** Rajeev Shrestha, Hyun Ji Lee, Junmo Lim, Pallavi Gurung, Til Bahadur Thapa Magar, Young-Tak Kim, Kija Lee, Seulgi Bae, Yong-Wan Kim

**Affiliations:** 1 Dongsung Cancer Center, Dongsung Biopharmaceutical, Daegu 41061, Republic of Korea; 2 Department of Veterinary Internal Medicine, College of Veterinary Medicine, Kyungpook National University, Daegu 41566, Republic of Korea; 3 College of Veterinary Medicine, Kyungpook National University, Daegu 41566, Republic of Korea

**Keywords:** Ce6, PDD, PDT, photosensitizer, canine tumors

## Abstract

This work aims to prepare pure Chlorin e6 (Ce6) and establish Ce6-mediated photodynamic therapy (Ce6-PDT) as a better therapy option for canine tumors as well as mouse tumor models. Five dogs suffering from various cancers were treated with Ce6-PDT from one to several times. After receiving the Ce6 (2.5 mg/kg) for 3 h, tumors were illuminated superficially or interstitially with 660 nm light. Two dogs underwent Ce6-guided fluorescence imaging by photodynamic diagnosis (PDD). Cell proliferation and apoptosis were detected by the 4,5-dimethylthiazol-2-yl)-2,5-diphenyl tetrazolium bromide (MTT) assay and western blot assay, respectively. Ce6-PDT efficacy was also determined using melanoma and pancreatic cancer mouse models. Two veterinary patients with mammary carcinoma and histiocytic sarcoma had their tumors significantly diminished and showed improved health after receiving Ce6-PDT. Moreover, in the cases of canine tumors, the adjunctive use of Ce6-PDD revealed cancers that were not visible with white light viewing and provided a visual contrast from surrounding tissues. Also, in vivo, Ce6-PDT remarkably reduced melanoma and pancreatic tumors in the mouse model. These findings could pave the way for a better understanding of the underlying processes of Ce6-PDT, making it an effective and safe candidate for use in human and veterinary applications to abolish various cancers.

## 1. Introduction

Over the past decades, photodynamic diagnosis (PDD) and therapy (PDT) have evolved as alternative non-invasive diagnostic and therapeutic modalities in the treatment of various oncological diseases [1,2]. PDD is the phenomenon of exhibiting fluorescence on tumor cells when a photosensitizer (PS) is administered to the patient followed by the utilization of visible blue light (405 nm) [3]. Notably, PDD has become a valuable technical tool during surgical operations because of its ability to distinguish tumors from adjacent normal cells [4]. PDT, on the other hand, entails the use of photosensitizers (PSs), as well as appropriate light irradiation (600–800 nm) and molecular oxygen [5]. During PDT, PS absorbs light and reaches a singlet excited state before shifting to an excited triplet state via a non-radiant intercrossing transition. In a Type 1 reaction, these species can excite biomolecules in their vicinity while transferring the additional energy to oxygen, which causes tissue injury by triggering the formation of reactive oxygen species (ROS) and peroxy radicals [6,7]. Meanwhile, in the Type II reaction, direct energy transfer occurs between PS and molecular oxygen in the triplet excited state, resulting in the production of singlet oxygen (^1^O_2_), which is a powerful oxidizer [8]. However, all pathways eventually lead to cell death by apoptosis, necrosis, and destruction of blood vessels, an effective strategy for cancer mortality [9]. Utilizing these mechanisms, PDT has successfully treated malignancies such as basal cell carcinoma [10], lung [11], pancreatic [12,13], oral [14], prostate [15], and bladder cancers [16].

PS is triggered by a light source with a certain intensity and wavelength, and hence serves as the crucial backbone for the PDD-PDT activity [17,18]. A study has shown that PS purity, amphiphilicity, pharmacological characteristics, target ability, and dosimetry estimation are all significant aspects when considering PDT efficiency in cancer treatment [19]. In this regard, researchers have developed PCs with high extinction coefficients in the red light region and high singlet oxygen quantum yields, such as 5-aminolevulinic acid, porphyrins, hemoglobin, chlorophyll, bacteriochlorophyll, etc. [20,21]. However, Chlorin e6 (Ce6) has been employed as a potent PS for PDT because of its activation by NIR light and relatively rapid elimination from the body with a higher efficiency of singlet oxygen generation. In addition, it also displays fluorescence qualities, which are a bonus for PDD [22]. Furthermore, according to Zhang et al., Ce6 has safety features in that it is a NIR fluorescent imaging dye that operates in the spectral range of 650–900 nm, avoiding light absorption and scattering by endogenous tissue chromophores in deep tissues [23]. Ce6′s revealing properties, such as low dark toxicity in the absence of light, ease of handling, high activation capacity, and high singlet oxygen yield, make it a viable PS option in PDT [24,25]. Owing to its therapeutic efficiency, Ce6 or its conjugates-mediated PDD and PDT have been introduced for the diagnosis and treatment of most cancers [26,27,28]. 

**Scheme 1:**
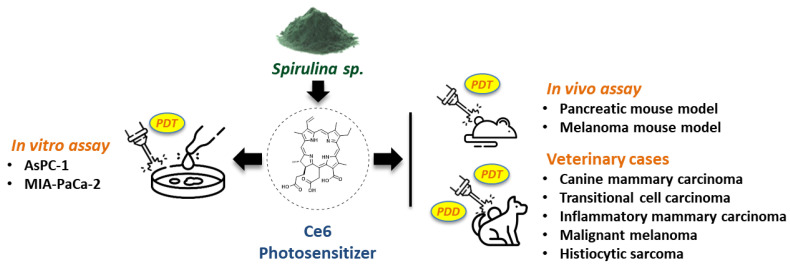
Synthesis of Ce6 and its PDT or PDD effects on veterinary cases, in vitro, and in vivo.

In the extension of photosensitizers as veterinary medicines, limited drugs such as EtNBS (5-ethylamino-9-diethylaminobenzo phenothiazinium chloride) [29], HPPH (Photochlor; 2-(1-hexyloxyethyl)-2-devinyl pyropheophorbid-a) [30], AlPcS4 (aluminiumphthalocyanine-tetrasulphonate) [31], mTHPC (m-tetrahydroxyphenylchlorine) [32], ZnPcS4 (Zinc phthalocyanine tetrasulfonate) [33], and 5-aminolevulinic acid (5-ALA) [34] have been described in various dogs with malignant tumors. However, there are no investigations into canine case studies of Ce6-PDT. We also reported the synthesis process of Ce6-curcumin conjugates as PS for PDT and evaluated their anti-pancreatic carcinoma activity as part of our ongoing Ce6 research [35]. Hence, our first objective was to synthesize highly pure Ce6 and study its PDT on in vitro and in vivo mouse models, while our main goal was to conduct canine case studies on five dogs with canine mammary carcinoma, transitional cell carcinoma, inflammatory mammary carcinoma, malignant melanoma, and histiocytic sarcoma, respectively (Scheme 1). Moreover, we also studied computed tomography (CT) scans of patients before and after the PDT to determine their detailed status. Ce6-PDD was also used to diagnose cancer in two dog cases by using the fluorescence bio imaging approach. 

## 2. Materials and Methods

### 2.1. Preparation of Ce6

The dried *Spirulina platensis* was purchased from EID PARRY INDIA LTD. *Spirulina platensis* was stirred in EtOH for 8 h in an N_2_ atmosphere to extract chlorophyll a, a valuable biomolecule fraction. It was then filtered, and the chlorophyll a-containing filtrate was washed with hexane and water. The hexane solution of chlorophyll a was treated with 1N HCl (pH 2–3) for 3 h at room temperature in an N_2_ atmosphere to obtain pheophytin a. For the neutralization, 1 N of NaOH was added to the reaction mixture. The portion of hexane containing pheophytin was purified by using liquid–liquid extraction, and hexanes were removed through a rotary evaporator. Thus, the obtained dry pheophytin a was dissolved in acetone and stirred for 10 min. Further, anhydrous sodium sulfate (Na_2_SO_4_) was added to the reaction, stirred for 30 min, and then filtered. The filtrate acetone was refluxed at 65–72 °C and the pH was maintained at 12.8 by adding 1N NaOH. The reaction mixture was refluxed for 6 h under inert conditions. The precipitated Ce6 was filtered, washed with acetone, and dried for 3 h in a vacuum oven (40 °C). The synthesized Ce6 was analyzed through NMR, ESI MS, IR, and HPLC [Supporting information (SI)–Sections S1–S5]. The formed Ce6 with PVP is coined as Phonozen^®^, Dongsung Biopharmaceutical, Seoul, Republic of Korea. 

### 2.2. Cell Culture

AsPC-1, MIA PaCa-2, and B16F10 cells were procured from the Korean Cell Line Bank (KCLB, Seoul, Republic of Korea) while Panc02 cells were obtained from the Gene target. AsPC-1, a human pancreatic cancer cell line, and Panc02, a mouse pancreatic cancer cell line, were grown in RPMI-1640 media (Life technologies corporation, Carlsbad, CA, USA) supplemented with 10% fetal bovine serum (life technologies corporation, USA) and 1% penicillin and streptomycin (Life technologies corporation, USA), whereas MIA PaCa-2, human pancreatic cancer cell line and B16F10, a murine melanoma cancer cell line, were grown in DMEM supplemented with 10% fetal bovine serum (Life technologies corporation, USA) and 1% Penicillin & Streptomycin (Life technologies corporation, USA). 

### 2.3. Cell Viability Assay

5 × 10^3^ cells were seeded in 96 well plates and incubated overnight at 37 °C in a CO_2_ incubator. The cells were treated with different concentrations of Ce6 (0, 0.03, 0.1, 0.3, and 1 mM) for 3 h. After 3 h, the cells were exposed to 660 nm, 50 mW, 5 J/cm^2^ laser light and were re-incubated for 72 h in the dark at 37 °C in a 5% CO_2_ incubator. Further, the MTT assay was used to determine cell viability. The absorbance at 540 nm was measured using a microplate reader (Multiskan GO, Thermo Fisher Scientific Oy, Ratastie 2, FI-01620 Vantaa, Finland).

### 2.4. Western Blot

Total protein was extracted by using radioimmunoprecipitation assay (RIPA) buffer (150 mM Sodium chloride, 1% Triton X-100, 0.5% Sodium deoxycholate, 0.1% Sodium dodecyl sulfate (SDS), and 50 mM Tris adjusted to pH 8.0) containing 1X protease and phosphatase inhibitors. The protein content was quantitated by using the Bradford protein assay kit. Sodium dodecyl sulfate-polyacrylamide gel electrophoresis (SDS PAGE) was used to separate protein samples, which were then transferred to Immobilon P membranes (Millipore Corp., Bedford, MA, USA) for up to 1 h. Blocking was done by using 5% skim milk in 1× Tris buffered saline (TBS) Tween-20 (TBS-T) at room temperature for 1 h. The membranes were then incubated at 4 °C overnight with primary antibodies againstmouse anti-Bcl-2 and rabbit anti-Bax (Bioss), rabbit anti-β-Actin (abcam) antibodies, and rabbit anti-caspase-3 (Cell signaling technology) antibodies. Specific bands were detected using an enhanced chemiluminescence (ECL) kit (Pierce, Rockford, IL, USA) under a luminescent image analyzer (Amersham, GE Healthcare, Chicago, IL, USA).

### 2.5. Mouse Model

Six-week-old male C57BL6 mice (n = 18) weighing 21 g were purchased from Orient Bio (Sungnam, Korea), and mice were housed in a standard environment (20 ± 2 °C; 50 ± 5% humidity; 12:12 h light/dark cycle, diet, and filtered water ad libitum) in the animal house facility of Dongsung Cancer Center, Daegu, for 7 days. Each experimental group consisted of randomly grouped mice of similar weight. All the mouse experiments were reviewed and carried out with the approval of the Institutional Animal Care and Use Committee of the Dongsung Cancer Center under protocol IACUC #ds002106117-2. The experiments were carried out in compliance with the ARRIVE guidelines.

### 2.6. Xenograft Mouse Model Using B16F10 Melanoma Cell Line

After a week of acclimation, 10 mice were chosen at random for the study. Mice received a subcutaneous injection of 0.1 mL of B16F10 cells (1 × 10^7^ cells/mL) into their right flanks. When the tumor mass reached the range of 90–100 mm^3^ after inoculation, a total of 10 tumor-bearing mice were randomly divided into control (untreated, n = 5) and Ce6-PDT-treated groups (n = 5).

### 2.7. Xenograft Mouse Model Using Panc02 Pancreatic Cell Line

Eight mice were injected subcutaneously with 0.1 mL of Panc02 pancreatic cells (1 × 10^7^ cells/mL) in their right flanks for the study. The tumor-bearing mice were randomized into two different groups; the control group (untreated) (n = 3) and the treatment group given Ce6 and laser irradiation (n = 5) when the volume of the tumor was in the range of 90–100 mm^3^.

### 2.8. PDT in Animal Model

Each mouse in the treatment group received an intravenous injection of 2.5 mg/kg Ce6. After 3 h, the right tumor was irradiated with a red light at a rate of 100 J/cm^2^ for 8 min 20 s. The tumor was measured with a vernier caliper, and the tumor volume (V) was calculated by using V = L × W^2^/2, where L and W were the length and width, respectively.

### 2.9. Veterinary Case Study

Between February 2020 and May 2022, five dogs were brought to the Kyungpook National University Veterinary Medical Teaching Hospital to be examined for clinical symptoms associated with various malignancies. The five subjects ranged in age from 5 to 14 years old and weighed between 4.0 and 9.3 kg. Table 1 shows the status of the patients, tumors, and concurrent therapy in detail. Histopathological analysis of the biopsy samples was used to diagnose each tumor. The owner of each client signed a written consent form for PDT. Surgery was performed in all patients before PDT or chemotherapy, and only chemotherapy was performed after PDT application.

### 2.10. PDD and PDT of Veterinary Case Study

A 2.5 mg/kg Ce6 IV injection was given to each patient 3 h before the PDD/PDT. The patients were examined under sedation or general anesthesia. All patients underwent general sedation with 5 µg/kg IV and 15 µg/kg IM injections of medetomidine, which dosed up to a total of 30 µg/kg. Similarly, before the application of interstitial PDT, butorphanol (0.2 mg/kg, IV) was administered once. After the PDT treatment, atipamezole (150 µg/kg) was given by IM injection for recovery from anesthesia. 

Before PDT, cases 4 and 5 were given PDD, for which patients received 2.5 mg/kg of Ce6 intravenously. After 3 h, the tumor areas were then irradiated with a 405 nm LED light. A bandpass filter was installed on the video camera (660 nm, Edmund Industrial Optics, Inc., Barrington, NJ, USA) to suppress blue light (for the observation of fluorescence). For fluorescence spectrometry, we used the DCC4K imaging system (Dongsung Biopharmaceutical, Republic of Korea) to obtain the spectra of each tissue. Ce6 had a maximum fluorescence peak at around 660 nm. 

For the PDT, a 660 nm laser diode (LD, LEMT, Belarus) with a radiation fluence of 200 mW/cm^2^ was used to irradiate the tumor. For superficial lesions, the quartz fiber with the macro lens (10 mm in diameter) was aimed towards the surface of the tumor, while an over-the-needle intravenous catheter (19 gauge needle) was inserted at the desired location in the tumor mass for the interstitial irradiation. A 1 cm flexible cylindrical diffuser fiber (a quartz core and polymer cladding; Taihan Fiberoptics Co., Ansan, Republic of Korea) was put in place via the catheter’s external cylinder, which had been left in place to support the fiber. Ce6-PDT was administered to patients five to ten times, depending on their responsiveness. Ce6-PDT treatment of all the dogs was carried out with the owners’ consent at the Kyungpook National University Veterinary Medical Teaching Hospital in Kyungpook.

## 3. Results

### 3.1. Absorbance and Fluorescence of Ce6

**Figure 1:**
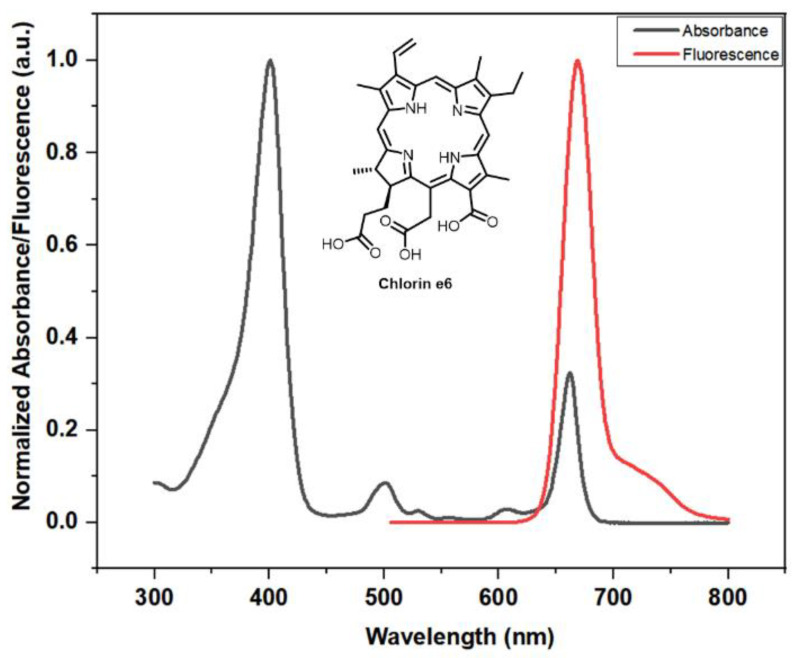
Absorbance and fluorescence of Ce6 in 95% EtOH solution (10 µM).

Ce6 has strong absorption peaks at wavelength of 402 and 662 nm, as well as exhibiting intense fluorescence at 668 ± 2 nm (Figure 1). This absorbed light has been useful for photochemical processes in PDT to generate ROS [36]. Moreover, as shown in Section S6, Figure S4 (SI), the ability of Ce6 to produce singlet oxygen, the key factor in the photodynamic killing of cancer cells, under photosensitizing conditions was studied using 1, 3-diphenylisobenzofuran (DPBF) as a singlet oxygen trap. The result showed the generation of ^1^O_2_ was enhanced with the increasing dose of Ce6 in comparison to groups with DPBF only.

### 3.2. In Vitro Cytotoxicity of Ce6-PDT in Pancreatic Cancer

**Figure 2:**
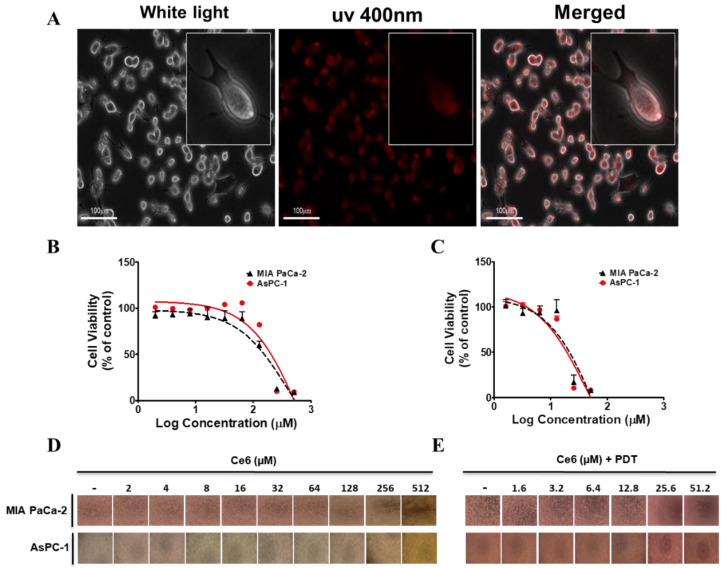
Ce6-mediated cytotoxicity on human pancreatic cancer cells. (**A**) Fluorescence images of MIA PaCa-2 cells after 24 h incubation with Ce6. The images shown are the white light images (left), 400 nm (middle), and merged of two images (right). The scale bar represents 100 μm. (**B**,**C**) Dark and photo-induced cytotoxic activity of Ce6 against AsPC-1 and MIA PaCa-2 cell lines (equivalent concentration 0–512 μΜ for dark and 0–51.2 μΜ of Ce6 for light). Medium with laser was taken as a control group. The data are the representation of mean ± SEM obtained from three independent experiments. (**D**,**E**) Morphological changes in cells after Ce6 treatment.

Antitumor effects of Ce6-PDT therapy on two pancreatic cancer cell lines, AsPC-1 and MIA PaCa-2, were investigated using a cell proliferation assay. The cellular uptake of Ce6 in MIA PaCa-2 cells was confirmed by using the fluorescence microscopic imaging technique (HyVolution, Leica microsystems) followed by 3 h incubation (Figure 2A). Cell viability of pancreatic cancer cells was measured after 72 h of treatment with Ce6 in the presence and absence of light. The results are shown in Figure 2B–E. At lower concentrations, AsPC-1 and MIA PaCa-2 had IC50 values of 19.7 and 21.75 µg, respectively. Ce6 alone, on the other hand, does not affect the viability of pancreatic cancer cells, indicating that Ce6 does not exhibit cytotoxicity at its lower dose. Furthermore, Ce6 dark toxicity caused a decrease in cell survival only when high doses were applied. 

Therefore, Ce6 alone required a higher concentration (more than 100 µg/mL) to elicit pancreatic cancer cell cytotoxicity, with an IC50 of 209.6 µg/mL and 155.2 µg/mL. This result indicates that normal cell uptake of Ce6 is slower, resulting in decreased toxicity, which is beneficial for PDT as its main purpose is to destroy only malignant cells.

### 3.3. Effects of Ce6-PDT on Cell Apoptosis of AsPC-1 and MIA PaCa-2 Cells 

**Figure 3:**
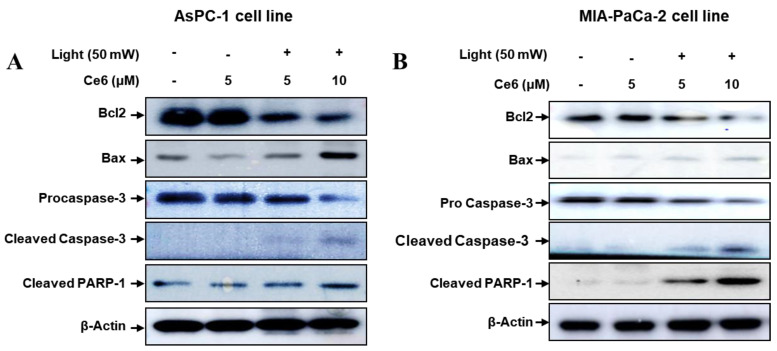
Ce6-mediated PDT effects in protein levels of apoptosis. AsPC-1 and MIA PaCa-2 cells were treated with Ce6 (5 and 10 μM) for 3 h followed by or without PDT. The western blot assay detected the protein levels of Bcl-2, Bax, Procaspase-3, cleaved caspase-3, and cleaved PARP-1 in (**A**). AsPC-1 and (**B**) MIA PaCa-2 cell line. β-Actin was used as a loading control.

The fundamental goal of PDT is to eliminate undesirable cells via ROS in various ways, one of which is apoptosis. Since caspases are early effectors for triggering PDT-mediated apoptosis, we examined the levels of Bax, caspase-3, and PARP-1 with anti-apoptotic Bcl-2 proteins in Ce6-PDT-treated AsPC-1 and MIA PaCa-2 cells (Figure 3A,B). Western blot analysis demonstrated that treatment with 5 and 10 μM of Ce6 in combination with PDT significantly reduced Bcl-2 protein expression while increasing apoptotic molecule expression of Bax compared to Ce6 only treatment. Furthermore, Ce6-PDT activated caspase-3 through its cleavage and upregulated the expression of cleaved PARP-1 in a dose-dependent manner. Taken together, the findings strongly suggest that Ce6-mediated PDT induces pancreatic cancer cell apoptosis via caspase-3 activation, resulting in tumor growth suppression.

### 3.4. In Vivo Effect of Ce6-PDT in Xenograft Mouse Model 

**Figure 4:**
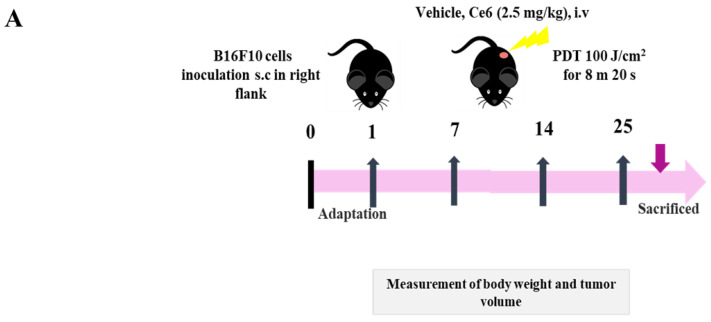

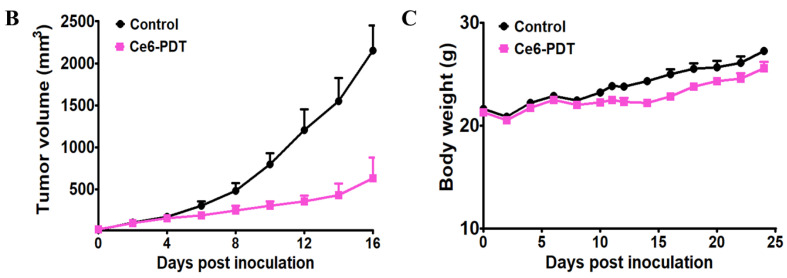
Anti-cancer activities of Ce6-PDT against melanoma allografts. (**A**) Timeline of the experimental procedure. The mice were subcutaneously injected at the right flanks with 0.1 mL of B16F10 cells (1 × 10^7^ cells/mL). The control group was not exposed to any treatment. (**B**) Changes in tumor volume and (**C**) body weight for control and treatment groups. Data are expressed as mean ± SEM, n = 5.

Since the Ce6-PDT demonstrated potential phototoxicity in vitro, we used a xenograft mouse model using B16F10 melanoma cells to investigate Ce6′s efficacy as a PDT agent in vivo. To explore the role of Ce6-PDT phototoxicity in vivo, C57BL/6 mice were transplanted with B16F10 cells for a tumor xenograft model (Figure 4A). Tumor-bearing mice from the vehicle control and Ce6-PDT groups were injected intravenously with vehicle (normal saline) and 2.5 mg/kg Ce6 solution, respectively. As shown in Figure 4B, tumor volume increased after the subcutaneous transplantation but was significantly reduced by subsequent Ce6-PDT treatment compared to the control group. However, as shown in Figure 4C, there were no body weight changes in the mice during the treatment period compared to the controls.

### 3.5. Xenograft Mouse Model Using PANC02 Pancreatic Cell

**Figure 5:**
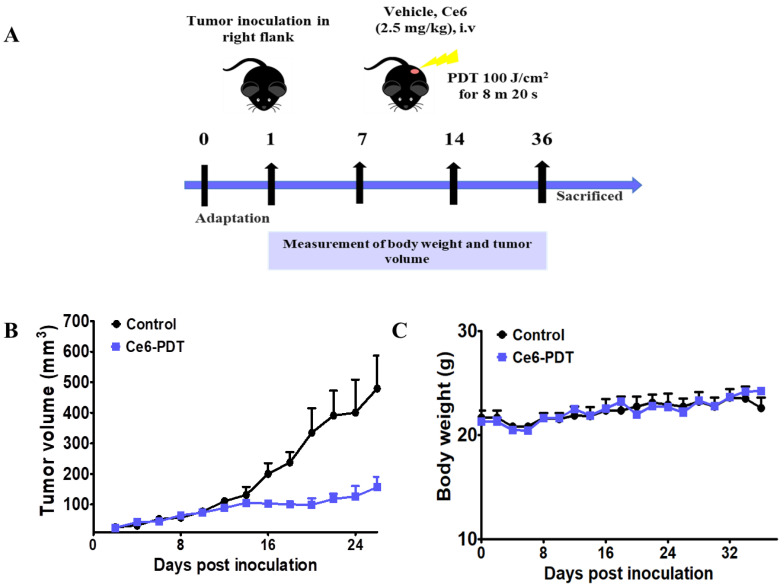
In vivo therapeutic effect in PANC02 tumor models. (**A**) Experimental scheme of tumor induction and Ce6-PDT treatment. (**B**) Tumor volume variations of each group over time. (**C**) Body weight variations of each group over time. Data are expressed as mean ± SEM, n = 5.

To further corroborate our in vitro findings, we generated a preclinical model of pancreatic cancer by using a PANC02 cell line in nude mice. The mice were intravenously administered with Ce6 (2.5 mg/kg), followed by laser irradiation (3 h post-injection), as shown in the experimental scheme (Figure 5A). In Ce6-PDT-treated mice, the laser irradiation site revealed considerable tumor necrosis, which was not seen in the vehicle-treated control mice. As shown in Figure 5B, Ce6-PDT inhibits tumor growth and improves anti-tumor activity. No significant changes in body weight were noticed between the vehicle-control and Ce6-PDT-treated groups (Figure 5C).

### 3.6. Photodynamic Diagnosis in Dogs 

**Figure 6:**
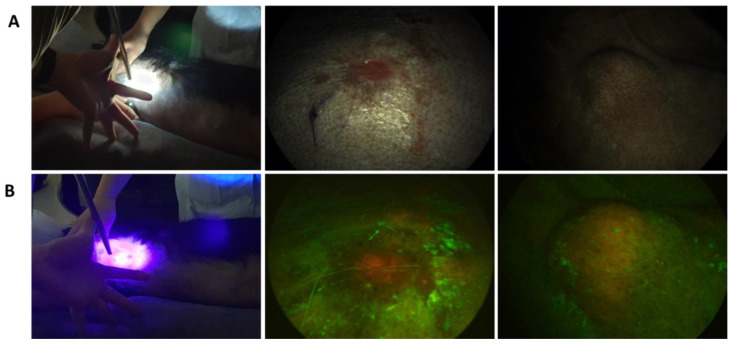
Photodynamic diagnosis (PDD) cases with representative images. The dog was intravenously injected with 2.5 mg/kg Ce6. After 3 h, prior to the PDT, fluorescence was detected in dog’s abdominal tumor and normal groin skin (**A**) with white light and (**B**) blue light at 405 nm. In the blue light, intense fluorescence was detected at tumor locations, whereas normal tissues had no fluorescence.

Ce6-PDD’s diagnostic accuracy was tested in two tumor cases (4 and 5). After receiving Ce6 (2.5 mg/kg), both patients demonstrated red fluorescence at specific tumor sites (Table 1). Figure 6 displays a representative image of red fluorescence caused following injection of Ce6 in the axillary skin, abdominal skin, and peripheral lymph nodes of dog 5. Under white light, the tumor in case 5 was hardly visible, making it difficult to identify it from normal tissues (Figure 6A). However, when using blue light with Ce6, it was apparent that it was not readily apparent under white light. The fluorescence detected in the digital camera images of the tumor and the surrounding normal tissues at 3 h after intravenous injection of Ce6 (2.5 mg/kg) revealed the following results. During irradiation with blue light, strong red fluorescence was released from only the abdominal tumor through the skin due to tumor-specific uptake of Ce6, giving considerable visual contrast from the surrounding normal tissues and the normal groin skin (Figure 6B). This fluorescence data confirms that Ce6, a photosensitizer, accumulates in higher amounts in tumor tissue than in normal tissue.

### 3.7. Photodynamic Therapy in Dogs 

**Figure 7:**
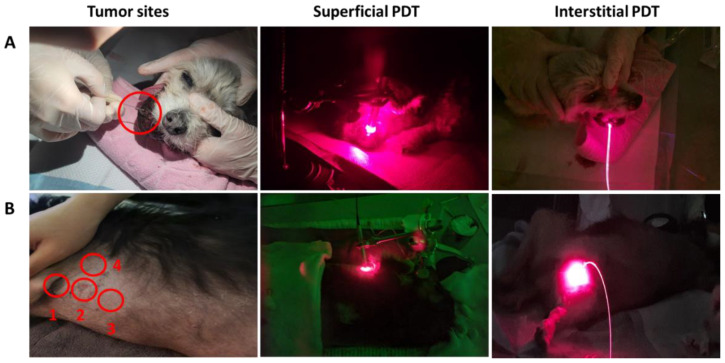
Photodynamic therapy of case no. 4 and 5. (**A**) PDT on case 4 at oral mucosa through the superficial and interstitial method. (**B**) PDT on case 5 at the abdominal and groin skin through the superficial and interstitial methods. The patients were intravenously injected with 2.5 mg/kg Ce6. After 3 h, the tumor was firstly treated with superficial irradiation by using the quartz fiber fitted with a macrolens which was set toward the surface of the tumor. For the interstitial irradiation, an over-the-needle intravenous catheter (19 gauge needle) was inserted at the desired location of the tumor mass. In both methods, the light irradiation was 660 nm and emitted (200 mW, 100 J) by a laser diode under general anesthesia. The treatment was processed for 500 s in repeated manners.

**Figure 8:**
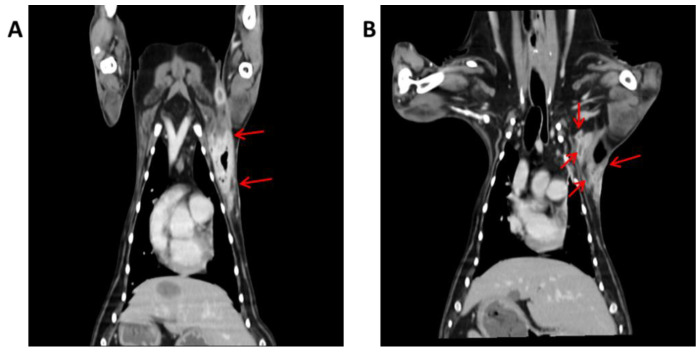
Computed tomography (CT) images of dog 5. Dog 5 was diagnosed with histiocytic sarcoma at left axillary skin, abdominal and groin skin before first PDT. (**A**) Opacities of tumors areas could be observed in the images of the axillary skin (red arrow), (**B**) After second PDT treatment, the tumor on axillary skin was significantly diminished in size and the margin of the mass became unclear.

In the further extension of Ce6-PDT, five patients diagnosed with different malignant tumors were represented as the study population. These dogs were treated with an intravenous injection of 2.5 mg/kg Ce6, and after 3 h, PDT was undertaken by delivering a red light superficially or interstitially at 200 mW. The demographic characteristics of these dogs are given in Table 2. The types of neoplasia in the study population included mammary carcinoma, transitional cell carcinoma, inflammatory mammary carcinoma, malignant melanoma, and histiocytic sarcoma. The first patient, the Shih Tzu dog, exhibited a good response after receiving superficial and interstitial PDT 10 times in a few months without getting any other treatments. The size of the subject’s tumor was reduced by 2.96 times after PDT, and the subject survived. Dog 5, with histiocytic sarcoma at several sites, was treated with multiple PDT at axillary skin lesion and groin lesion with enlarged peripheral lymph nodes (Figure 7B). On the CT scan after the second PDT application, the size of the mass on the axillary skin lesion decreased from 9.9 × 9.95 × 15.3 to 9.6 × 5.9 × 11.1 mm significantly, and the margin of the mass became unclear. The mass on the groin lesion was maintained. In the course of PDT treatment, simultaneously, chemotherapy was also conducted in this patient. The distinct suppression of tumors was also analyzed through the comparison of CT scans before and after the PDT (Figure 8A,B), while CT scans of tumors in other cases (cases 2–4) are provided in the supplementary information (SI Sections S7–S10).

In the Maltese dog with transitional cell carcinoma (dog 2), the Bichon Frise dog with inflammatory mammary carcinoma (dog 3), and the mixed dog with malignant melanoma (dog 4), Ce6-PDT did not result in positive therapeutic outcomes (Figure 7A), because these subjects were advanced in age and the tumors had already metastasized or were at the final stage of tumor progression. Dogs 3, 4, and 5 visited our hospital in a stage of recurrence after surgical resection at other hospitals; consequently, the growth of the tumors was very rapid, and the response to chemotherapy was poor compared to other patients. 

## 4. Discussion

Ce6 is a second-generation photosensitizer and is predominantly used in the treatment of various cancers including lung, bladder, skin, as well as head and neck cancers [37]. On the other hand, this is the first research employing Ce6-associated PDT in the treatment of canine tumors that we are aware of. Second, Ce6-PDT’s advantage in our study was its potential ability to target a variety of cancers, such as pancreatic and melanoma, that have demonstrated minimal response to chemotherapy. Because Ce6 is a potent photosensitizer with good stability and a 660 nm absorbance wavelength [24,25], it was chosen for this study. In this study, we synthesized ultrapure Ce6 for tumor imaging and photodynamic therapy and studied its characteristic absorbance and fluorescence. 

Because one of the most significant properties of a photosensitizer is its strong biocompatibility, the cytotoxic effects of Ce6 and its PDT in pancreatic cancer cell lines, AsPC-1, and MIA PaCa-2 cells were investigated. Briefly, pancreatic cells were exposed to Ce6 (2–512 μM) in the dark and Ce6 (2–51.2 μM) with irradiation at 660 nm (50 mW, 5 J/cm^2^) for 3 h. In the absence of radiation, Ce6 was shown to have low toxicity, while there was a considerable decline in viability under radiation, which is attributable to PDT. The findings show that Ce6 has a modest cytotoxic impact in the dark, but when irradiated with light of 660 nm wavelength, it activates cytotoxic species. This, together with a lower IC50 value (19.7, 21.75 µM) could enhance therapeutic efficacy and suppress Ce6 resistance. A growing body of evidence has revealed that the rapid phototoxic effect arises from various mechanisms, including apoptosis and necrosis [6,7,8,9]. As a result, the markers of apoptosis were analyzed by western blot to investigate the involvement of Ce6-mediated PDT in apoptosis. According to Yu et al., Ce6-mediated photodynamic action on lung cancer cells is preceded by DNA damage through elevated intracellular ROS levels [38]. Our data showed that Ce6-PDT treatment induced apoptosis in AsPC-1 and Mia PaCa2 cells, as evidenced by increased Bax expression, and decreased Bcl-2 expression. PDT treatment significantly enhanced the formation of cleaved caspase-3 and cleaved PARP-1 in both AsPC-1 and MIA PaCa2 cells. This finding is in line with our earlier studies, which reported the impact of Ce6-curcumin conjugates on pancreatic cancer up-regulated apoptotic markers [35]. These data suggest that Ce6-PDT treatment inhibits proliferation and promotes apoptosis of pancreatic cancer cells.

Motivated by the positive response of Ce6-PDT in vitro, we evaluated its influence on tumor growth in vivo using B16F10 and Panc02 cells xenografted into mice. Tumors in the control groups developed rapidly during the treatment period, whereas tumors in the Ce6-PDT group grew slowly, demonstrating that Ce6-PDT can prevent tumor growth. Thus, cancer cell proliferation was significantly reduced in Ce6-PDT-treated heterotopic Panc02 xenografts compared to controls. Ce6-PDT was also successful in treating melanoma tumors grown heterotopically. However, the body weights of the PDT group did not vary significantly in comparison with the control groups. Hahn et al. confirmed our findings, showing that carbon dot-Ce6-hyaluronate conjugate PDT was successful in treating melanoma skin cancer [26]. In addition, Ce6 nanoparticles have also been studied as a potential therapy for pancreatic cancer [39,40]. The majority of Ce6-associated research has been done in combinations, although research with only Ce6 as a photosensitizer for cancer therapy is scarce.

Tumor treatments remain a major challenge in veterinary medicine. PDT has been widely employed in the treatment of many tumors because of its less intrusive effect and low side effects. As previously mentioned, different photosensitizers have been utilized therapeutically in veterinary medicine for PDT [29,30,31,32,33,34,41,42]. However, Ce6-mediated PDD for diagnostic reasons and PDT are less well-known in veterinary medicine, hence, we executed Ce6-PDD and PDT with single or multiple treatments on five different canine tumor patients. Compared to previous studies, our study conducted PDT treatment on fewer patients and was less effective in treating malignant tumors in dogs. Only two dogs (dogs 1 and 5) showed a positive response to PDT. Case 1, which was the dog with mammary carcinoma, showed a gross reduction in mass size. In dog 5, a decrease in tumor size was observed in the CT images, but it is difficult to attribute this response to PDT because this patient was controlled with chemotherapy and PDT simultaneously. It was also difficult to observe a positive response to PDT in the remaining three patients, perhaps because PDT treatment was initiated when the tumor was already too advanced or had recurred in patients after surgery. However, the value of this study is that Ce6-PDT was very safe in dogs and did not have any noticeable side effects. Some patients had bleeding at the sites after PDT application, but it recovered quickly, and there were no skin lesions except erythema. The diagnostic process of Ce6-PDD prior to PDT in cases 4 and 5 revealed red fluorescence imaging, which clearly distinguished tumor tissue from normal tissues. Thus, if further study is conducted, Ce6-PDT can be considered as a new diagnostic tool and management method for malignant tumors in dogs. 

## 5. Conclusions

In summary, we have synthesized the highly pure second-generation photosensitizer Ce6. This investigation established that Ce6-PDT significantly inhibited cell proliferation of AsPC-1 and MIA PaCa2 through downregulation of Bcl-2 and upregulation of bax, cleaved PARP-1, and cleaved caspase-3, thus triggering an apoptotic pathway. Moreover, it effectively suppressed the tumor growth not only in in vivo melanoma and pancreatic mouse models but also in canine tumors. Therefore, the present study inaugurates that Ce6-PDT will be an effective and safe candidate for human and veterinary applications to abolish different types of cancer. Meanwhile, the demonstration of Ce6-PDD mediated fluorescence imaging of malignant melanoma and histolytic sarcoma reveals a safe, alternative, and efficient diagnostic way for the diagnosis of cancer treatment. The medical application of Ce6-mediated PDT/PDD and its relevance to the prediction of clinical outcomes in veterinary medicine should be explored further based on the implications of the study. 

## Figures and Tables

**Table 1:** Summary of the subjects treated with photodynamic therapy using Ce6.

No.	Species	Breed	Age (y)	Sex	Weight (kg)	Tumor Type	Tumor Site When PDT Application	Simultaneous Treatment
1	Dog	Shih Tzu	11	F	5	Canine mammary carcinoma	Rt. Mammary gland	-
2	Dog	Maltese	14	M	4	Transitional cell carcinoma	Urinary bladder	Chemotherapy (Mitoxantrone)
3	Dog	Bichon Frise	12	F	5.5	Inflammatory mammary carcinoma	Mammary gland	Chemotherapy (Cyclophosphamide, Mitoxantrone)
4	Dog	Mixed	11	M	5.5	Malignant melanoma	Rt. Mandible, oral mucosa	Surgery (Rt. total hemimandibulectomy, Rt. submandibular lymphadenectomy), Chemotherapy (Carboplatin, Imatinib)
5	Dog	Dachshund	5	M	9.3	Histiocytic sarcoma	Axillary skin lesions, abdominal and groin skin, peripheral lymph nodes	Surgery, Chemotherapy (Doxorubicin, CCNU, Imatinib)

**Table 2:** Application of PDT using Ce6. The light fluence rate and energy fluence were 200 mW/cm^2^ and 100 J/cm^2^.

Case No.	Light	No. of Treatment		Outcome
	Equipment	Superficial	Interstitial	
1	Laser diode	5	5	Alive
2	Laser diode	3	0	Died by Metastasis
3	Laser diode	7	5	Died by Metastasis
4	Laser diode	6	1	Died by Metastasis
5	Laser diode	5	7	Alive

## Data Availability

All the data are contained within the manuscript and Supplementary Materials.

## Supplementary Materials

The following supporting information can be downloaded at: https://www.mdpi.com/article/10.3390/life12122102/s1, Section S1: General procedure; Section S2: Characterization data of Ce6; Section S3: ^1^H NMR spectra of Ce6; Section S4: Copy of ESI mass of Ce6; Section S5: HPLC spectra of Ce6; Section S6: Singlet oxygen study; Section S7: Copy of Computed tomography (CT) scans of case-1; Section S8: Copy of Computed tomography (CT) scans of case-2; Section S9: Copy of Computed tomography (CT) scans of case-3; Section S10: Copy of Computed tomography (CT) scans of case-4.
