# Supplementary material for: Effect of Photodynamic Therapy with Chlorin e6 on Canine Tumors
Source: Life (Basel). 2022 Dec 14;12(12):2102. doi: 10.3390/life12122102 (PMC9782963; doi:10.3390/life12122102)
Supplement: Supplementary file 1 [file life-12-02102-s001.zip › life-2064042-supplementary.pdf]

Supplementary Information

Effect of photodynamic therapy with Chlorin e6 on canine tumors

Rajeev Shrestha[1,†], Hyun Ji Lee[2,†], Junmo Lim[1], Pallavi Gurung[1], Til Bahadur Thapa Magar[1], Young-Tak Kim[1], KiJa Lee[3], Seulgi Bae[2,*], and Yong-Wan Kim[1,*]

[1]Dongsung Cancer Center, Dongsung Biopharmaceutical, Daegu, South Korea

[2]Department of Veterinary Internal Medicine, College of Veterinary Medicine, Kyungpook National University, Daegu, South Korea.

[3]College of Veterinary Medicine, Kyungpook National University, Daegu, South Korea.

[†]These authors contributed equally to this work.

*Correspondence: sgbae@knu.ac.kr, thomas06@hanmail.net

TABLE OF CONTENTS

Section S1. **General information**

Section S2. **Characterization data of Ce6**

Section S3. **^{1}H NMR spectra Ce6**

Section S4. **Copy of ESI mass of Ce6**

Section S5. **HPLC spectra of Ce6**

Section S6. **Singlet oxygen study**

Section S7. **Copy of Computed tomography (CT) scans of case-1**

Section S8. **Copy of Computed tomography (CT) scans of case-2**

Section S9. **Copy of Computed tomography (CT) scans of case-3**

Section S10. **Copy of Computed tomography (CT) scans of case-4**

Section S1. General information

Waters Alliance HPLC system (Waters, Houston, TX, USA) consisting of a binary pump, an online degasser and a diode array detector (DAD) was used to determine the contents of flavonoids. For chromatographic analysis, a Capcell C18/X-terra (4.6mm × 250 mm, 5μm) column was used at room temperature. The mobile phase consisted of 0.08% TFA water (A) and acetonitrile (B) using a gradient program of 45–100% (B) in 0–20 min, 45–55% (B) in 20-30 min, 45-55% (B) in 30-35 min. All the solutions were filtered through 0.22 μm to the HPLC analysis and monitored at 407 nm. The mass spectra were recorded in Waters micromass Z_Q by electronspray ionization (ESI) in the positive and negative mode. The parameters were as follows: capillary voltage: 2.50 KV; cone voltage: 40 V; extractor voltage: 3 V; RF lens voltage: 0.3 V; source temperature: 100 ºC; desolvation temperature: 200 ºC; desolvation gas flow rate: 350.0 L/h; cone gas flow rate: 50.0 L /h; scanning range: from 100 to 1,000 amu. ^{1}H NMR and ^{13}C NMR spectra were recorded on DPX Bruker (600 MHz) spectrometers in deuterated DMSO using the solvent chemical shift of 2.5 ppm and water peak at 3.35 ppm. Fourier transform infrared (FT-IR) spectra were recorded on PerkinElmer FT-IR spectrometer Spectrum Two^{TM}. Absorbance spectra of the tested compound was recorded at room temperature (298 K) using UV/Vis spectrophotometer (Thermo-scientific, Skanlt software 5.0). The sample was prepared in 95% ethanol solvent (Duksan, HPLC grade pure) at a concentration of 10 μM. The data was corrected for solvent background by the instrument's calibration using the 95% ethanol as a blank. The absorption spectra of sample in solution was obtained in the range of 300-800 nm at 1 nm interval in 3 determination using three trial samples. The fluorescence (Tecan-Spark) measurement was carried out at room temperature at 405 nm (excitation wavelength).

Section S2. Characterization data of Ce6

Ce6 :Black powder; ^{1}H NMR (DMSO-d_6, 600 MHz): δ 14.0 (1H, s), 12.4 (2H, s), 9.7 (1H, s), 9.6 (1H, s), 9.1 (1H, s), 8.2 (1H, m), 6.3 (1H, dd, J =16.8,1.2 Hz), 6.1 (1H, dd, J =12.0,1.2 Hz), 5.4-5.3 (2H, m), 4.6 (1H, dd, J =14.4,7.2 Hz), 4.5 (1H, dd, J =13.2, 2.4 Hz), 3.8-3.7 (2H, m), 3.6 (3H, s), 3.5 (3H, s), 3.2 (3H, s), 2.7-2.6 (1H, m), 2.4-2.3 (1H, m), 2.2-2.1 (1H, m), 1.7-1.63 (6H, m), 1.61-1.5 (1H, m), -1.6 (1H, s), -1.9 (1H, s). Ir: 3303, 2961, 1695, 1565, 1438, 1384, 1304, 1236, 1164, 1028, 962 cm^{-1}. ESI MS [H^+]: 597.

Section S3. ^{1}H NMR spectra Ce6

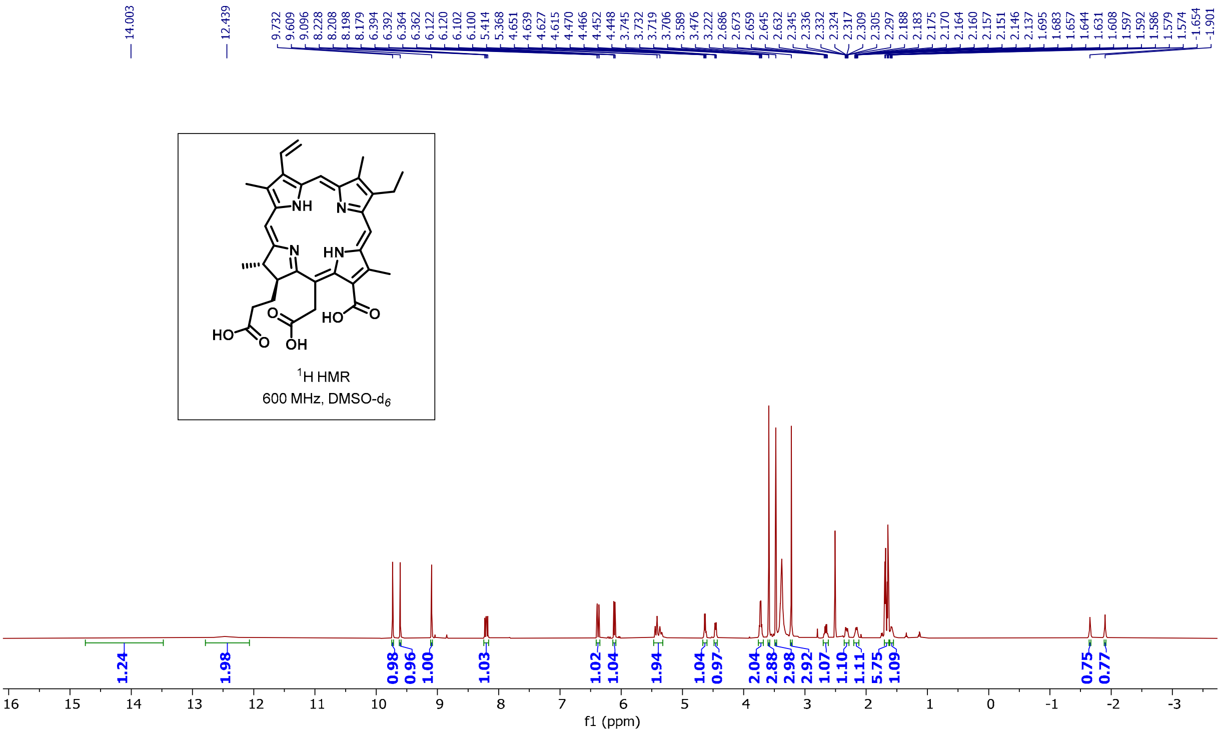

Figure S1. ^{1}H NMR of Ce6. NMR was analysed in solvent DMSO-d_6.

Section S4. Copy of ESI mass of Ce6

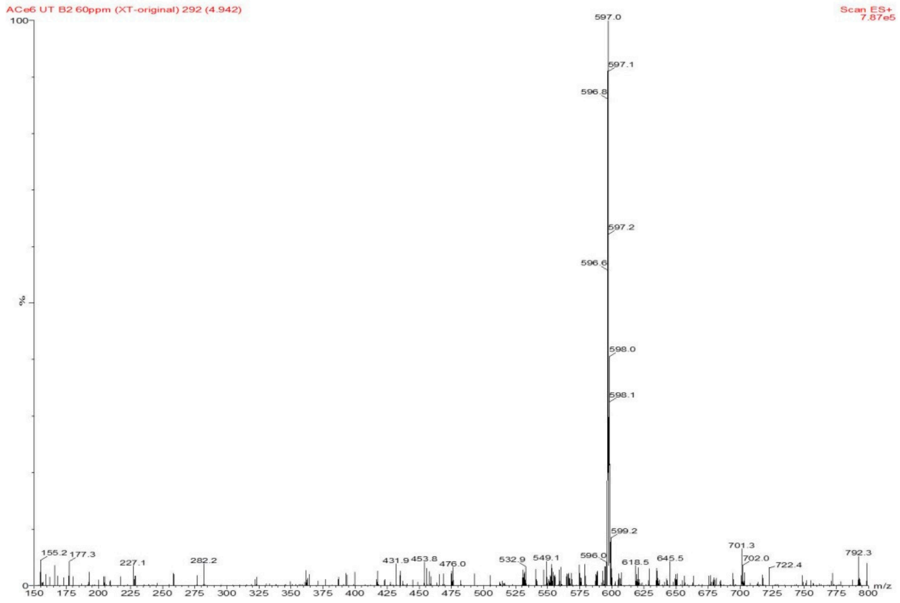

Figure S2. Positive ESI-MS of Ce6.

Section S5. HPLC spectra of Ce6

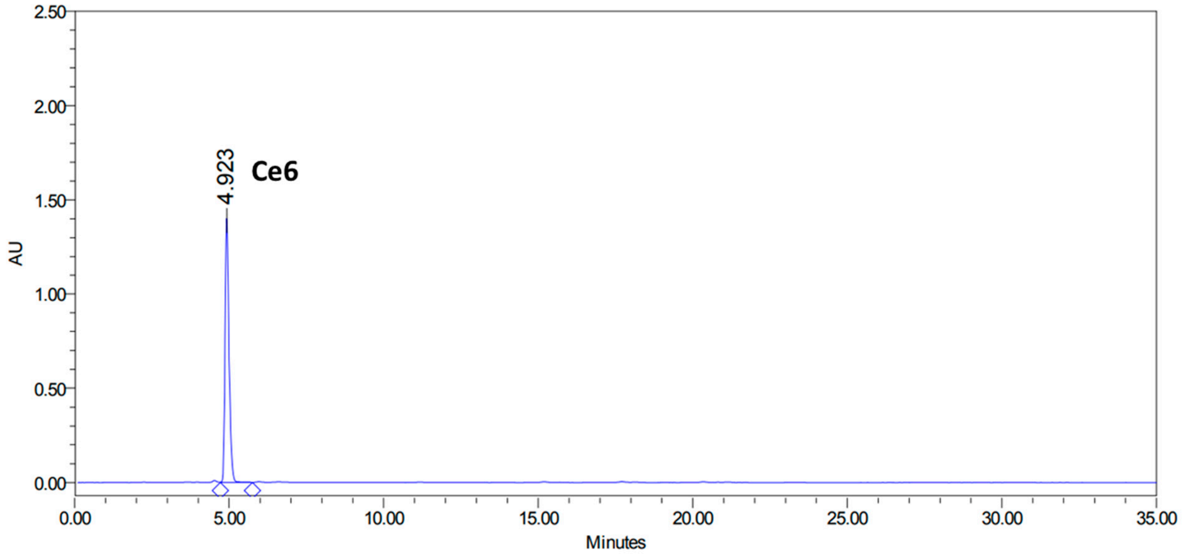

Figure S3. HPLC chromatogram of Ce6 at wavelength 407 nm.

Section S6. Singlet oxygen study

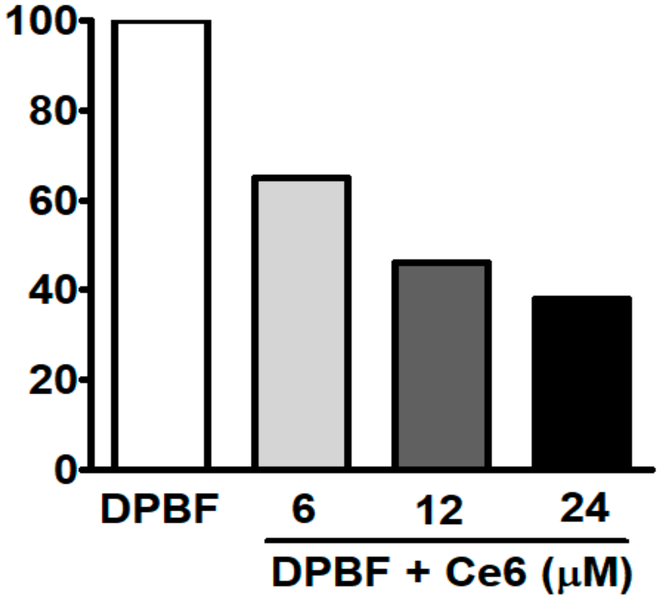

Figure S4. Absorbance decay (%) of Ce6 followed by photo-irradiation in dose-dependent manner. Solutions of DPBF solution only (50 μM) or Ce6 (6, 12, and 24) μM with DPBF were placed in a 96-well plate and the container was covered with aluminum foil and were irradiated with light of 50 mW, 5 J/cm^2 for 40 s. After irradiation, visible spectra of the sample solutions were measured spectrophotometrically. The data are expressed as mean of three experiments.

Section S7. Copy of Computed tomography (CT) scans of case-1

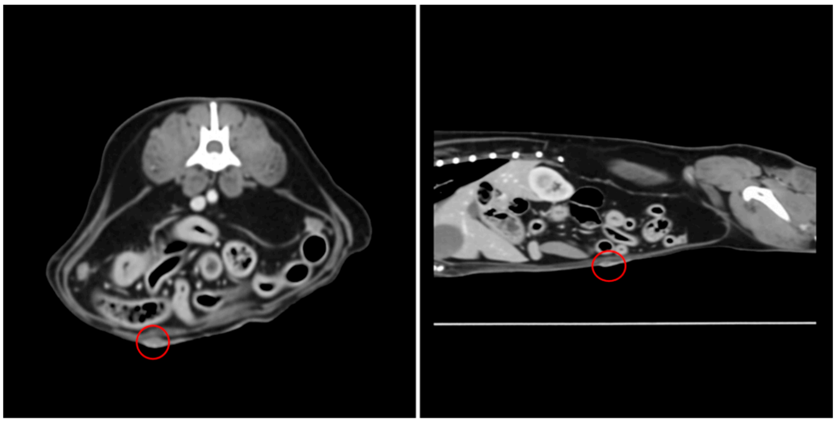

Figure S5. Computed tomography (CT) images of case No. 1. The case-1 dog, Shih Tzu was diagnosed with canine mammary carcinoma at right mammary gland before 1st PDT. Opacities of soft tissue could be observed in the images of the mammary gland (red circle).

Section S8. Copy of Computed tomography (CT) scans of case-2

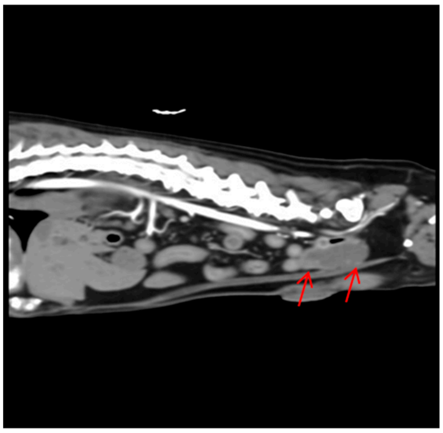

Figure S6. Computed tomography (CT) images of case No. 2. The case-2 dog, Maltese was diagnosed with transitional cell carcinoma at urinary bladder before 1st PDT. Opacities of soft tissue could be observed in the images of the mammary gland (red arrows).

Section S9. Copy of Computed tomography (CT) scans of case-3

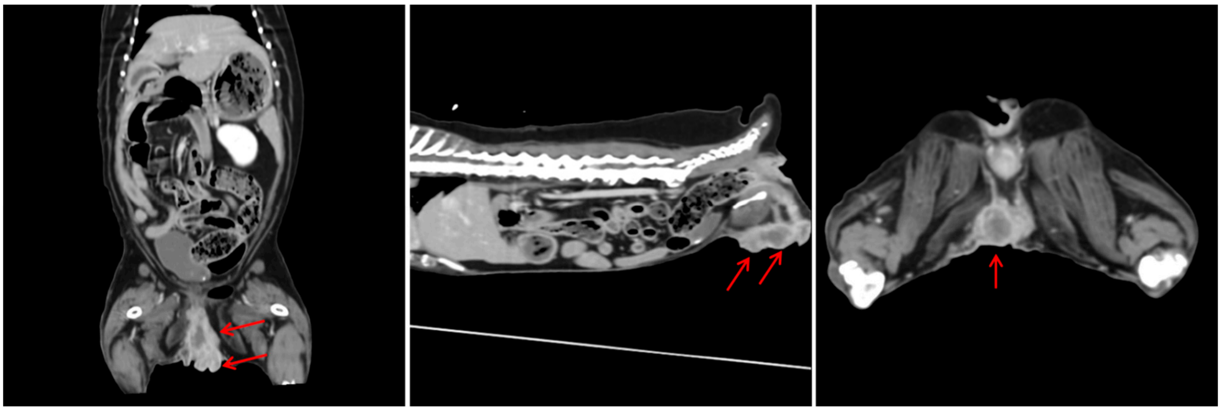

Figure S7. Computed tomography (CT) images of case No. 3. The case-3 Bichon Fries dog was diagnosed with canine inflammatory mammary carcinoma at mammary gland before 1st PDT. Opacities of soft tissue could be observed in the images of the mammary gland (red arrows).

Section S10. Copy of Computed tomography (CT) scans of case-4

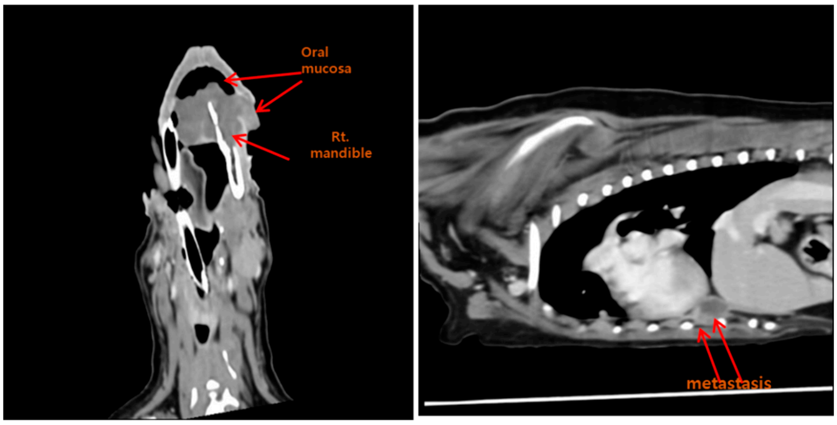

Figure S8. Computed tomography (CT) images of case No. 4. The case-4 Castrated dog was diagnosed with malignant melanoma at rt. Mandible and oral mucosa before 1st PDT. Opacities of soft tissue could be observed in the images of the rt. Mandible and oral mucosa (red arrows).
